# Christian Science

**Published:** 1913-02

**Authors:** 


### Christian Science

A while ago we attended a Christian Science lecture, not because we are tending in that direction nor "investigating" the subject, but because it is a good plan for everyone to hear the other side rather than a reiteration of his views, whether they be political, religious or philosophic. In one sense, the strength of any cult lies in its audience. It must be admitted that Christian Science is phenomenally strong in this respect. This audience was large, decorous, thoughtful, if not keenly and analytically critical. One little thing impressed us forcibly, we met several friends and were introduced to several others deeply interested in the cause. There was not a sign of the vulgar gloating over the presence of one of a different faith that is to be seen at many meetings, political, religious and medical, and no hair-trigger proselyting such as mars the dignity of some churches. In other words, these people were too well bred hosts to express surprise at the presence of a guest. However, we may disagree with Christian Scientists, they are almost without exception gentlemen and ladies, and we like them in social life because they are not eternally fussing about their insides and tempting us to talk shop about their viscera, as do so many of our lay adherents.

The speaker, Prof. Hermann S. Hering, C. S. B., of the Board of Lectureship of the "Mother Church" in Boston, stated the prime moving cause of Christian Science to be a desire to heal disease and sin, and a dissatisfaction with the established medical and theologic efforts in these respective directions. Without attempting to speak for the theologians, we feel that we represent every conscientious physician in saying that no organization is more keenly and actively dissatisfied with the practical results of medicine than the medical profession itself. That we have gained signal victories in many instances, only makes us more anxious to achieve the same success in others. The fact that apparently fixed barriers have been passed by our therapeutic measures, always based on clearer understanding or material demonstrations—has done away with the old complacence that this or that condition was humanly speaking irremediable. And very few of us would be restrained by prejudice from adopting any method shown to be of value. Indeed, except in conception and what appears to us an unwarranted generalization, essentially the methods of Christian Science have long been in use by the medical profession and are prominent in its therapeutics today.

At the outset of the lecture, it occurred to us that the lecturer's own words were inconsistent with his expressed views. But it soon became apparent that this inconsistency was precisely the converse of what would be noticed in a lecture by a materialist and due to the fact that language has been built up by man recognizing both matter and spiritual force.

It was stated that physical scientists themselves have relegated matter to a basis of force or energy by demonstrating that the atom is composed of electrons, each of which is a unit of positive or negative electric energy. The whole logic of the speaker is well illustrated by this sophistry. Neither the electron nor the atom is an established fact but merely a plausible and probable theory. And no physical scientist of whom we know, has confused the energy manifested in the putative electron, with its material substance. Instead of the ionic theory abandoning the conception of matter for energy, it has rather tended to restore the older conception of electricity as a stream of actual particles.

Whatever may be said of "spirit"—and our disinclination to discuss this point implies no disbelief in the conception—practically all forms of science, in all times, have clearly recognized both matter and force as distinct concepts, fundamentally axiomatic. There is no demonstration of an axiom. To use the words of the lecturer, 2x2=4 always and everywhere, and the man who declares the product to be 5 is radically wrong. But we do not know how to convince him of his error, nor, indeed, how to convince the Christian Scientist that he is the one who is putting down 5 as the product. But we have known of otitis media, gastric ulcer and chronic typhlitis convincing individual Christian Scientists that they had been doing so. Indeed, our own copy of Science and Health was presented as a material token of such conviction.

